# Neuroprotective Effect of Ligustilide through Induction of α-Secretase Processing of Both APP and Klotho in a Mouse Model of Alzheimer’s Disease

**DOI:** 10.3389/fnagi.2017.00353

**Published:** 2017-11-02

**Authors:** Xi Kuang, Hong-Jing Zhou, Amy H. Thorne, Xi-Nan Chen, Lin-Jiao Li, Jun-Rong Du

**Affiliations:** ^1^Key Laboratory of Drug Targeting and Drug Delivery System, Department of Pharmacology, West China School of Pharmacy, Sichuan University, Chengdu, China; ^2^Ludwig Institute for Cancer Research, University of California, San Diego, La Jolla, CA, United States

**Keywords:** Ligustilide (LIG), Klotho, APP, α-secretase processing, β-amyloid

## Abstract

Emerging evidence suggests that alpha-processing single transmembrane proteins, amyloid precursor protein (APP) and anti-aging protein Klotho, are likely to be involved in the progression of Alzheimer’s disease (AD). The natural phthalide Ligustilide (LIG) has been demonstrated to protect against aging- and amyloid-β (Aβ)-induced brain dysfunction in animal models. The present study is to investigate the effects of LIG on cognitive deficits and metabolism of both APP and Klotho and its underlying mechanism in AD double-transgenic (APP/PS1) mice and cultured human cells. Our results show that treatment with LIG significantly ameliorated memory impairment and Aβ levels and plaques burden. Specifically, LIG might act as a potent enhancer of α-secretase, disintegrin, and metalloprotease 10 (ADAM10), leading to upregulation of alpha-processing of both APP and Klotho and subsequent increases in the levels of both soluble APP fragment (sAPPα) and soluble Klotho (sKL) with inhibition of IGF-1/Akt/mTOR signaling in AD mice and cultured cells. Moreover, the specific ADAM10 inhibitor (G1254023X) effectively reversed LIG-induced alpha-processing of both APP and Klotho *in vitro*, while Klotho gene knockdown by small interfering RNA significantly blunted LIG-mediated inhibition of IGF-1/Akt/mTOR signaling *in vitro.* Taken together with the reported neuroprotective effects of both sAPPα and sKL as well as autophagy induction by Akt/mTOR pathway inhibition, our findings suggest that neuroprotection of LIG against AD is associated with induction alpha-processing of APP and Klotho and potential Aβ clearance. Whether LIG might induce Aβ autophagic clearance and the underlying mechanisms need to be further studied.

## Introduction

Alzheimer’s disease (AD) is a neurodegenerative disease characterized by progressive memory loss. Extracellular senile plaques and intracellular neurofibrillary tangles are the main neuropathological hallmarks of AD (Xie et al., 2017). Amyloid beta (Aβ) is a 39–43 amino acid peptide that makes up the core of senile plaques and these Aβ oligomers have been shown to play a central role in the progression of AD (Walsh et al., 2002).

Amyloid beta is proteolytically produced from a single transmembrane amyloid precursor protein (APP), which can be cleaved through two different pathways: amyloidogenic and non-amyloidogenic. Cleavage through the amyloidogenic pathway occurs by the proteolytic enzymes beta and gamma secretase at the N and C termini, and results in the release of Aβ into the extracellular space (Kowalska, 2004). Cleavage through the non-amyloidogenic pathway involves the activation of α-secretase, a member of the disintegrin and metalloprotease (ADAM) family, which cleaves APP within the sequence of the Aβ domain. This action precludes the generation of the Aβ peptide and releases a soluble APP fragment, sAPPα (Lopez-Font et al., 2015). The sAPPα fragment has been shown to have both neurotrophic and neuroprotective effects (Chasseigneaux and Allinquant, 2012; Harti et al., 2013).

Aging of the central nervous system (CNS) is one of greatest risk factors in the development of neurodegenerative pathologies such as AD. Thus, maintaining a healthy neuronal population for as long as possible is a promising therapeutic strategy to combat neurodegeneration of the aging brain (Mohajeri et al., 2015). There have been several reports describing the proteins and signaling pathways involved in regulating the aging process. For example, Klotho is a putative aging-suppressor gene which encodes a type-I transmembrane protein. It is predominantly expressed in the distal convoluted tubules of the kidney and the choroid plexus of the brain, with weaker expression in the pituitary and parathyroid glands, as well as the hippocampus (Kuro-o et al., 1997; German et al., 2012). Similar to the other type-I-transmembrane proteins such as APP and Notch, Klotho is processed by α-secretase (ADAM10 and ADAM17) as well as β and γ-secretases (Bolch et al., 2009). During its processing, the shed ectodomain of Klotho is released into the blood and cerebrospinal fluid and also named as soluble Klotho (sKL). Accumulating evidence indicates that this sKL may play important roles as a neuroprotective factor, thereby supporting healthy brain aging (Hensel et al., 2016; Cararo-Lopes et al., 2017). While it has been shown that mutation of Klotho leads to systemic aging and reduced longevity in mice (Kuro-o et al., 1997; Shiozaki et al., 2008), increasing Klotho expression has been shown to promote healthier aging and prolongation of life (Kuro-o, 2011).

Ligustilide (LIG; 3-butylidene-4,5-dihydrophthalide) is the main lipophilic constituent of the *Umbelliferae* family of medicinal plants, including *Radix angelicae sinensis* and *Ligusticum chuanxiong*, and readily crosses the blood-brain barrier (Chen et al., 2010). Our previous studies have shown that LIG exerts marked neuroprotective effects against several CNS pathologies including forebrain ischemic injury in ICR mice, permanent forebrain ischemia in rats, focal cerebral ischemia/reperfusion in rats, scopolamine induced memory impairment in mice, hydrogen peroxide-induced injury in PC12 cells, and lipopolysaccharide-induced inflammation in primary rat microglia (Kuang et al., 2006, 2008, 2014a; Yu et al., 2008; Wang et al., 2010; Cheng et al., 2011).

In this study, we examined the effect of LIG on cognitive impairment in the APP/PS1 double-transgenic mouse model of AD. Specifically, we investigated the mechanisms underlying the efficacy of this compound, including α-secretase catalytic activities, APP processing, and signaling pathway regulation by the sKL protein.

## Materials and Methods

### Animals and Treatments

For the production of LIG, a well-established procedure in our laboratory was followed as previously described (Kuang et al., 2006). Male transgenic mice APPswe/PS1dE9 [B6/J-Tg (APPswe, PSEN1dE9) 85Dbo/Nju], hereafter “APP/PS1”, and C57 BL/6 mice, hereafter “C57”, were purchased from Beijing HKF Bioscience Co. Ltd. APP/PS1 mice were intragastrically administered either LIG (30 mg/kg, in 3% Tween 80) or vehicle control (3% Tween 80 alone) for 14 weeks (*n* = 12 per group), starting at 8.5 months of age. C57 mice were administered vehicle control only (*n* = 12). Treatments were administered by oral gavage one time each day and body weights were recorded weekly. After behavioral testing was completed, the mice were killed by a sodium pentobarbital overdose (50 mg/kg, i.p.) and perfused through the heart with cold saline. The brain was removed: one hemisphere was frozen in liquid nitrogen and stored at -80°C until further analysis, and the other hemisphere was fixed in 4% paraformaldehyde for 24 h, dehydrated through an ethanol series (70, 95, and 100%), penetrated with chloroform, and embedded in paraffin. All procedures were approved by the Animal Research Committee of West China School of Pharmacy. All animal studies were conducted in accordance with the Regulations of Experimental Animal Administration issued by the State Committee of Science and Technology of the China.

### Y-Maze Test

Short-term working memory of mice was assessed using a Y-maze alternation task as previously described (Cheng et al., 2011). Briefly, each mouse was placed at the end of one arm and allowed to move freely throughout the maze over the course of 5 min. The series of arm entries was recorded visually. Alternation was defined as the mouse entering different arms of the maze in succession as a result of consecutive arm entering. Each successive non-overlapping entrance sequence (e.g., ABC, CAB, or BCA, but not BAB) was defined as one alternation. The percentage of spontaneous alternations was calculated by dividing the total number of alternations by the total number of entries minus 2, and the percentage of alternation was used as an index of short-term memory (Hiramatsu et al., 2010).

### Step-Down Passive Avoidance Test

Long-term working memory of mice was assessed using a step-down type of passive avoidance task as previously described (Hiramatsu et al., 2010). Briefly, each mouse was placed on a wooden platform (4 cm × 4 cm × 4 cm). When the mouse stepped down from the platform and placed all of its paws on the grid floor, an electric shock was delivered to the grid floor for 15 s. A retention test was carried out 24 h after the training session in a manner similar to the training, except that no electric shock was delivered to the grid floor. Each mouse was placed on the platform again and the latency to step-down was recorded. An upper cut off time of 300 s was set.

### Immunohistochemistry

Five micrometer sections of mouse brain were mounted on glass slides. The primary antibodies used in the study are summarized in **Table 1**. The sections were incubated with antibody diluted in PBS at 37°C for 3 h and then 4°C overnight. Immunoperoxidase staining was performed with an avidin-biotin complex kit (Boster Biological Technology). Immunoreactions were visualized using 3,3′-diaminobenzidine tetrahydrochloride and nuclei were lightly counterstained with Meyer’s hematoxylin (except NeuN staining). Negative control sections were only stained with secondary antibody to control for non-specific background. Semi-quantitative analysis of the immunostaining results was performed for three sections of each brain, with each section containing three microscopic fields from the cerebral cortex, hippocampal CA1 areas, and the choroid plexus for Klotho. The stereotaxic locations of the area selected was from bregma –1.34 to –1.94 mm. Immunoreactivity was determined based on the integrated optical density (IOD) of immunostaining per field using Image Pro Plus 6.0.

**Table 1:** Primary antibodies used in this study.

Antibody	Host	Dilution	Application	Source
Aβ1-42	Rabbit	1:100	IHC	Abcam
Klotho	Rat	1:100	IHC	Abcam
NeuN	Rabbit	1:100	IHC	Millipore
APP	Mouse	1:100	IHC	Boster
Akt	Rabbit	1:1000	WB	CST
p-Akt	Rabbit	1:1000	WB	CST
mTOR	Rabbit	1:1000	WB	CST
p-mTOR	Rabbit	1:1000	WB	CST
IGF-1R	Rabbit	1:500	WB	CST
p-IGF-1R	Rabbit	1:500	WB	CST

### Measurements of ADAM Enzymatic Activity

The method used for measuring ADAM enzymatic activity is based on the cleavage of a secretase-specific peptide conjugated to a fluorescent reporter molecule. Briefly, the brain tissue of APP/PS1 mice was homogenized in RIPA buffer (Beyotime, Jiangsu, China) by a hand-held motor and kept on ice for 1 h. The homogenates were centrifuged at 12,000 × *g* at 4°C for 20 min and supernatants collected. Lysate protein concentrations were adjusted to 2 mg/ml and lysates were then incubated with the fluorogenic ADAM-peptide substrate Mca-Pro-Leu-Ala-Gln-Ala-Val-Dpa-Arg-Ser-Ser-Ser-Arg-NH_2_ (10 μM, final concentration; R&D Systems) for 20 min at 37°C. Secretase cleavage results in a fluorescent signal that can be measured on a microplate reader (excitation wavelength 355 nm and emission wavelength 510 nm) and corresponds to the level of secretase enzymatic activity in each sample.

### Western Blot Analysis

For the tissue-based assay, the brains of three mice in each group were homogenized in RIPA buffer (Beyotime, Jiangsu, China) by a hand-held motor and kept on ice for 30 min to lyse the cells completely. The homogenates were then centrifuged at 14,000 × *g* at 4°C for 15 min. The supernatants were collected and protein concentration was determined using a BCA protein assay kit (Boster, Wuhan, China). Equal protein was mixed with 5 × SDS-PAGE sample loading buffer (Beyotime, Jiangsu, China) and boiled for 5 min at 99°C.

For the cell-based assay, cells were lysed with RIPA buffer (Beyotime, Jiangsu, China) according to the manufacturer’s instructions. Protein concentration was determined by a BCA protein assay kit (Boster, Wuhan, China) and equal protein concentrations were mixed with 5 × SDS-PAGE sample loading buffer (Beyotime, Jiangsu, China) and boiled for 5 min at 99°C.

All tissue and cell samples were fractionated using 10% SDS-PAGE and transferred to a PVDF membrane (Millipore). Membranes were incubated with primary antibodies overnight at 4°C, detected with HRP-conjugated secondary antibodies (Zhongshan Jinqiao Biology) and developed using an ECL chemiluminescence kit (Millipore). The optical density (OD) of each band was determined using Gel Pro Analyzer 6.0 (Media Cybernetics, Bethesda, MD, United States).

### Real-Time Quantitative PCR Analysis (RT-PCR)

Total RNA was isolated from either mouse brains or cells using TRIZOL reagent (Invitrogen) and cDNA was synthesized using Revert Aid First Strand cDNA Synthesis Kit (Ferments, Vilnius, Lithuania). RT-PCR was performed using SsoFast Eva Green Supermix (Bio-Rad, Hercules, CA, United States) according to the manufacturer’s instructions. The cycling conditions were as follows: initial denaturation at 95°C for 30 s, followed by 40 reaction cycles (95°C for 5 s, 60°C for 10 s, and 72°C for 10 s). The specific primer pairs (Invitrogen Trading, Shanghai, China) are listed in **Table 2**. Gene expression was normalized to β-actin and results are expressed as a fold change of the threshold cycle value relative to control using the 2^-ΔΔC_t_^ method (Livak and Schmittgen, 2001).

**Table 2:** The specific primer pairs used in polymerase chain reaction.

Gene	RNA Sequence	Sense	Antisense	Product
huKlotho	NM_004795.3	gctctcaaagcccacatactg	gcagcataacgatagaggcc	104 bp
huAPP	NM_201414	accgctgcttagttggtgag	ggtgtgccagtgaagatgag	113 bp
huβ-actin	NM_001101.3	ggagcccgtcggtaattttaa	tctgcatgtgcggttggtt	382 bp
msKlotho	NM_013823.2	tggggtcccattggatagag	actcagggtagtcgccgtc	129 bp
msAPP	NM_007471.2	ccgttgcctagttggtgagt	gctcttctcgctgcatgtc	142 bp
msADAM10	NM_007399.3	catgatgactactgcttggcctat	gcaccgcatgaaaacatcaca	1011 bp
msADAM17	NM_001277266.1	gctgaacctaaccccttgaag	ttagcactctgtttctttgctgtc	1839 bp
msβ-actin	NM_00739.3	ctgaccctgaagtaccccattgaaca	ctggggtgttgaaggtctcaaacatg	198 bp

### ELISA Assay

The levels of soluble and insoluble Aβ in the brains of APP/PS1 mice were quantified as previously described (Deng et al., 2006). Briefly, brains were homogenized in 4 × TBS buffer (w/v) and then centrifuged at 8000 × *g* for 1 h. The supernatants were used for measuring soluble Aβ. To measure insoluble Aβ, the tissue pellets were re-suspended in 5 M guanidine (2×) and sonicated. Homogenates (20 μl) were diluted with 10 × loading buffer and centrifuged at 8000 × *g* for 30 min; supernatants were loaded for ELISA. The soluble and insoluble Aβ levels in all samples were determined using the commercially available mouse Aβ40 and Aβ42 ELISA kit (CUSABIO) according to manufacturer’s instructions. Data obtained from brain homogenates is expressed as pg/mg total protein.

To determine the level of sKL and soluble APP (sAPPα) in the brains of APP/PS1 mice, tissue was homogenized in 10 × PBS and then stored at -20°C overnight. Cell membranes were disrupted through two freeze-thaw cycles and then centrifuged at 5000 × *g* for 5 min. Supernatants were used to measure sKL and sAPPα with the commercially available ELISA kits (CUSABIO) according to manufacturer’s instructions.

For the cell-based assays, cells were incubated with 1 or 5 μM LIG in DMSO for 24 h and then collected and centrifuged at 3,000 rpm, 4°C for 30 min. The supernatants were used to measure sKL and sAPPα by ELISA (CUSABIO).

### Cell Culture, Transfections, and Drug Treatments

Ligustilide was prepared as a 1 mM stock solution in DMSO and diluted with cell culture media before use. The final concentration of DMSO in the culture medium was 0.1%, which had no effect on cell viability. The same condition of DMSO was also added in controls during the cell treatment.

HEK293T cells were obtained from the American Type Culture Collection (ATCC, Manassas, VA, United States) and cultured in Dulbecco’s Modified Eagle’s Medium (DMEM; Gibco, Grand Island, NY, United States) supplemented with 10% fetal bovine serum (FBS; Hyclone, Logan, UT, United States) in a humidified atmosphere of 95% air and 5% CO_2_ at 37°C. For detection of cell viability, the cells were incubated with LIG (1 or 5 μM) for 24 h. For detection of sKL by ELISA and full-length Klotho by RT-PCR, cells were pre-incubated with either TAPI-O (10 μM, Santa Cruz, CA, United States) or G1254023X (10 μM, Sigma, St. Louis, MO, United States) for 30 min and then treated with LIG (5 μM) plus inhibitor for 24 h.

Klotho knock-down in HEK293T cells was achieved using siRNA. Briefly, proliferating cells were transfected with 10 μM control siRNA or Klotho siRNA (Santa Cruz Biotechnology) using Lipofectamine 2000 (Invitrogen, Carlsbad, CA, United States) according to the manufacturer’s instructions. To determine cell viability, cells were incubated with LIG at 5 μM for 24 h following transfection. To measure Klotho gene expression or protein expression, cells were incubated with LIG at 5 μM for 24 or 48 h following transfection, respectively.

Human neuroblastoma SH-SY5Y cells were obtained from the ATCC (Manassas, VA, United States). Cells were maintained in DMEM (Gibco, Grand Island, NY, United States) supplemented with 10% FBS (Hyclone, Logan, UT, United States) and cultured in a humidified atmosphere of 95% air and 5% CO2 at 37°C. SH-SY5Y cells were transiently transfected with mutant human APP695 cDNA (App_swe_, kindly provided by Rong Zhang, the Third Military Medical University) using Lipofectamine 2000 (Invitrogen) according to the manufacturer’s instructions. Cells were grown until nearly confluent, washed with serum-free medium, and then incubated with LIG at 5 μM for 24 h prior to the detection of cell viability. Prior to measuring sAPPα by ELISA and full-length APP gene expression by RT-PCR, cells were pre-incubated with G1254023X (10 μM) for 30 min and then treated with LIG at 5 μM plus inhibitor for 24 h.

### Cell Viability

The viability of cultured SH-SY5Y and HEK293T cells was measured using an MTT colorimetric assay (Sigma). Briefly, cells were plated overnight in a 96-well plate and then treated with LIG (0.1, 1, 5, and 10 μM) or control for 24 h, respectively. MTT was added to each well at a final concentration of 0.5 mg/ml and cells were incubated for 4 h at 37°C. The culture medium was removed and DMSO was added to each well to dissolve the resulting formazan crystals. The absorbance was measured at a wavelength of 570 nm using a microplate reader (Bio-Tek Instruments). Percent viability was defined as the relative absorbance of treated versus untreated control cells.

### Statistical Analysis

The data are expressed as mean ± SEM. Statistical analyses were performed using SPSS V.16.0. The data were analyzed using one-way ANOVA for repeated measures followed by a Tukey *post hoc* test. Values of *p* < 0.05 were considered statistically significant.

## Results

### LIG Treatment Improves Memory Deficits and Ameliorates AD-Induced Neuronal Loss in APP/PS1 Mice

It has been described that APP/PS1 mice exhibit early AD symptoms at around 6–7 months of age, which worsen within another 3 or more months (Reiserer et al., 2007). Therefore, we opted to treat mice with LIG for 14 weeks starting at 8.5 months of age. During this time, we found no significant effect of drug treatment on mouse weight compared to vehicle treated mice, indicating a lack of toxicity (data not shown).

**FIGURE 1:**
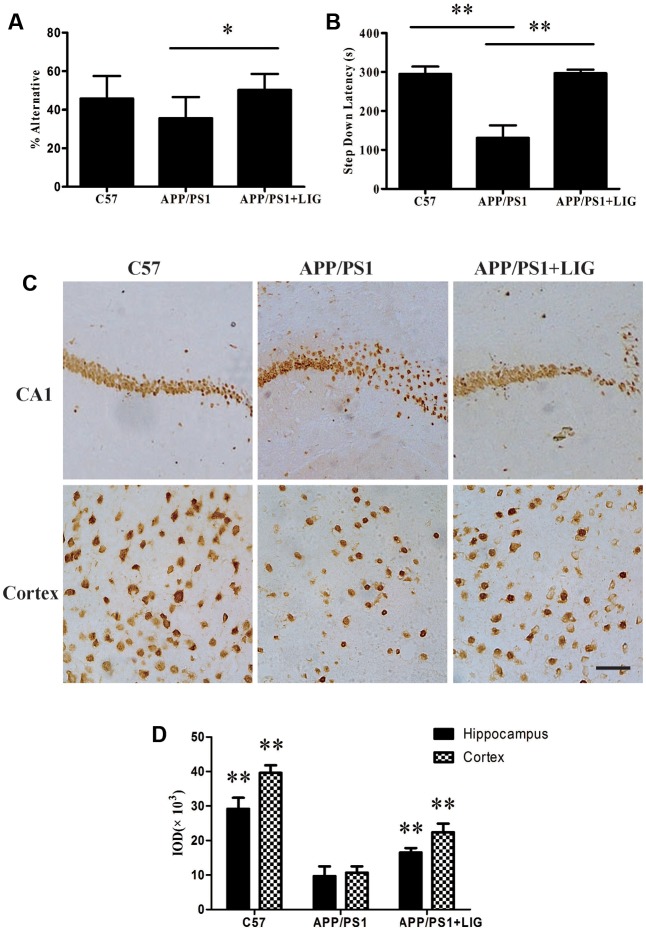
Effect of Ligustilide (LIG) on memory impairment and neuronal loss in amyloid precursor protein (APP)/PS1 transgenic mice. C57 mice were treated with vehicle control and APP/PS1 mice treated with either vehicle control or LIG (5 μM) for 14 weeks starting at 8.5 months of age. **(A)** Effect of LIG on Y-maze test. LIG treatment significantly increased the percentage of alternations used as an index of short-term memory. **(B)** Effect of LIG on the passive avoidance test. LIG treatment increased the step-down latency in comparison to vehicle-treated APP/PS1 mice. **(C)** Representative immunostaining of NeuN in the hippocampal CA1 region and cerebral cortex. Scale bar, 25 μm. **(D)** Quantitative image analysis of NeuN based on the integrated optical density (IOD) of positive immunostaining. The data are expressed as mean ± SEM; *n* = 12 for behavior tests, *n* = 4 for immunohistochemistry; ^∗^*p* < 0.05, ^∗∗^*p* < 0.01.

To investigate the effect of LIG on short- and long-term working memory, APP/PS1 mice were evaluated in a Y-maze spontaneous alternation task and a step-down passive avoidance task. As expected, the percentage of alternation in the Y-maze test was reduced in the vehicle-treated APP/PS1 mice compared to the wild-type C57 mice, indicating a reduction in short-term memory. Interestingly, chronic administration of LIG (30 mg/kg, 14 weeks) significantly rescued this effect, indicating a significant enhancement in short-term memory (*p* < 0.05; **Figure 1A**). Similarly, vehicle-treated APP/PS1 mice showed a significant reduction in step-down latency during the passive avoidance test compared to the C57 group (*p* < 0.01), and this effect was rescued in mice treated with LIG (*p* < 0.01; **Figure 1B**), indicating a significant enhancement in long-term memory. These results show that LIG therapy significantly improves the memory deficits seen in APP/PS1 mice.

Consistent with our behavioral test results, immunohisto chemistry analyses revealed a marked decrease in neuronal staining in both the hippocampal CA1 region and the cerebral cortex in vehicle-treated APP/PS1 mice compared to the wild-type C57 mice (**Figure 1C**). Interestingly, this loss was rescued following chronic LIG administration in the aged APP/PS1 mice compared to the vehicle-treated APP/PS1 mice (**Figures 1C,D**), with neuronal staining at a similar level as the C57 control group. Collectively, these results imply that LIG therapy improves short- and long-term memory through its prevention of neuronal loss in the APP/PS1 mouse model of AD.

### LIG Increases α-Secretase Catalytic Activity by Increasing ADAM10

**FIGURE 2:**
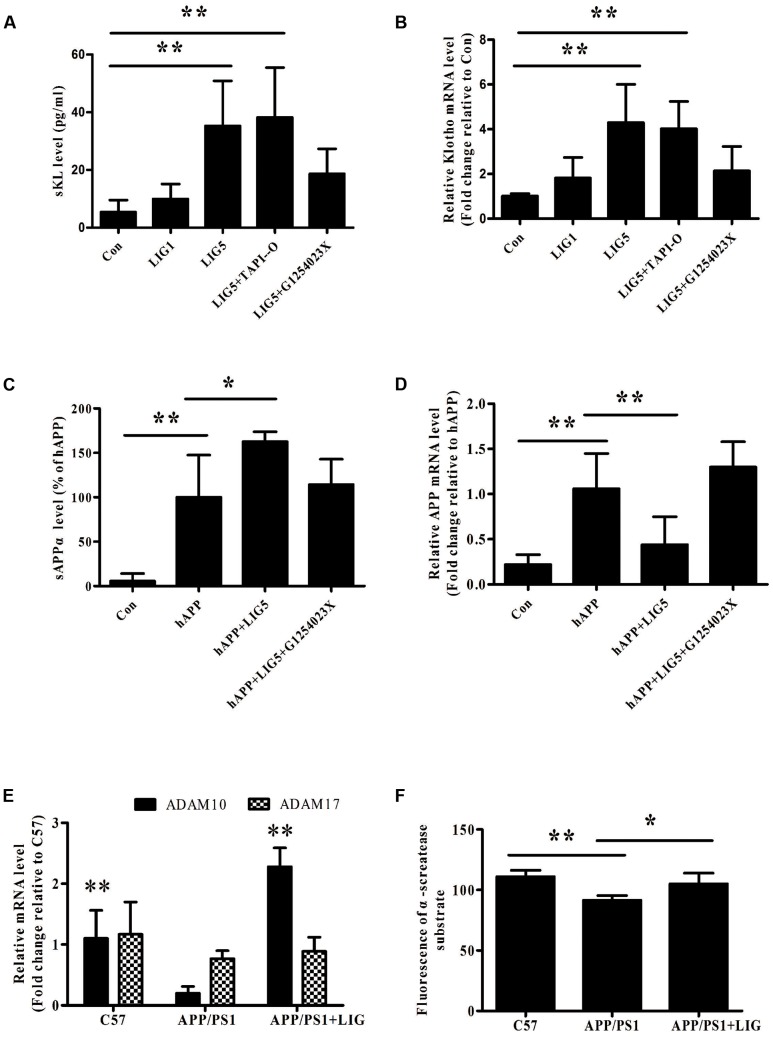
Effect of LIG on α-secretase catalytic activity *in vitro* and *in vivo*. **(A,B)** Effect of LIG (1 or 5 μM) on **(A)** soluble Klotho (sKL) secretion as measured by ELISA and **(B)** full-length Klotho gene expression as measured by RT-PCR in HEK293T cells. Effect of α-secretase processing was determined by culturing cells in the presence or absence of inhibitors specific to ADAM enzymes (TAPI-O, ADAM17 inhibitor; G1254023X, ADAM10 inhibitor). **(C,D)** Effect of LIG (1 or 5 μM) on **(C)** sAPPα secretion as measured by ELISA and **(D)** full-length APP gene expression as measured by RT-PCR in SH-SY5Ycells expressing hAPP. Effect of α-secretase processing was determined by culturing cells in the presence or absence of ADAM10 inhibitor G1254023X. **(E)** Effect of LIG on ADAM10 and ADAM17 gene expression in APP/PS1 mice as measured by RT-PCR. **(F)** Effect of LIG on ADAM enzymatic activity in APP/PS1 mice as measured by the fluorescence of an ADAM peptide-specific fluorescent reporter substrate. The data are expressed as mean ± SEM; *n* = 4 for RT-PCR, *n* = 6 for biochemical analysis in mice, and *n* = 8 for analyses in HEK293T and SH-SY5Y cells; ^∗^*p* < 0.05, ^∗∗^*p* < 0.01.

Our results thus far have demonstrated that LIG therapy attenuates cognitive impairment and reduces neuronal loss. To determine the effect of LIG on APP and Klotho processing, we first investigated the effect of LIG on Klotho and APP gene expression as well as its effect on sKL and sAPPα secretion by RT-PCR and ELISA, respectively. Using HEK293T cells and genetically modified SH-SY5Y cells engineered to express the human APP695 (hAPP), we show that LIG treatment plays a significant role in both the secretion and gene expression of these proteins. **Figures 2A,B** show that LIG treatment of HEK293T cells results in a dose-dependent increase in sKL secretion and Klotho gene expression. Interestingly, although LIG treatment significantly increased sAPPα secretion in the SH-SY5Y cells, we found a striking reduction in expression of the full-length gene (**Figures 2C,D**), indicating an increase in APP processing in the presence of LIG. Cell viability was examined after 24 h of incubation with LIG at 0.1–10 μM and we saw no effect of LIG (**Supplementary Figure S1**). Thus, LIG is non-toxic to HEK293T cells and hAPP-transfected SH-SY5Y cells.

Studies have shown a significant role for the ADAM family of enzymes in the catalysis and ectodomain shedding of APP, Notch, and Klotho among other cell surface proteins (Allinson et al., 2003; Bolch et al., 2009). To understand how LIG may be affecting the relative contributions of ADAM enzymes, we examined ADAM10 and ADAM17 in this study, as they have been shown to be relevant in AD (Lammich et al., 1999; Vingtdeux and Marambaud, 2012). RT-PCR analysis revealed a significant reduction in ADAM10 gene expression in APP/PS1 vehicle-treated control mice compared to the C57 control mice, with no effect on ADAM17 gene expression. Interestingly, we found a strong induction of ADAM10 gene expression in APP/PS1 mice following chronic administration of LIG, with little effect on ADAM17 (**Figure 2E**). To further investigate the functional relevance of ADAM10 in this model, we measured α-secretase activity in the brains of mice using a secretase-specific peptide conjugated to a fluorescent reporter molecule (see “Materials and Methods”). **Figure 2F** shows a significant increase in α-secretase activity in APP/PS1 mice treated with LIG compared with vehicle-treated APP/PS1 group (*p* < 0.05), indicating a role for LIG in ADAM10 α-secretase activity.

Thus far we have shown that LIG treatment increases Klotho and APP processing and also increases ADAM10 expression and enzymatic activity. Next, we used inhibitors specific to ADAM10 and ADAM17, G1254023X and TAPI-O, respectively, to examine the role of these enzymes in LIG-induced Klotho and APP processing. **Figures 2A,B** show that when HEK293T cells are pre-treated with G1254023X to inhibit ADAM10 prior to LIG there is a significant reduction in LIG-induced sKL secretion and Klotho gene expression, while inhibition of ADAM17 had no effect. Additionally, inhibition of ADAM10 resulted in a significant decrease in sAPPα, while rescuing APP gene expression in the hAPP-tranfectedSH-SY5Y cells (**Figures 2C,D**). Collectively, the results from these studies indicate that the effect of LIG on α-secretase enzymatic activity and downstream Klotho and APP processing is mediated by ADAM10.

### LIG Reduces Aβ Levels and Directs APP Processing toward the Non-amyloidogenic Pathway in APP/PS1 Transgenic Mice

**FIGURE 3:**
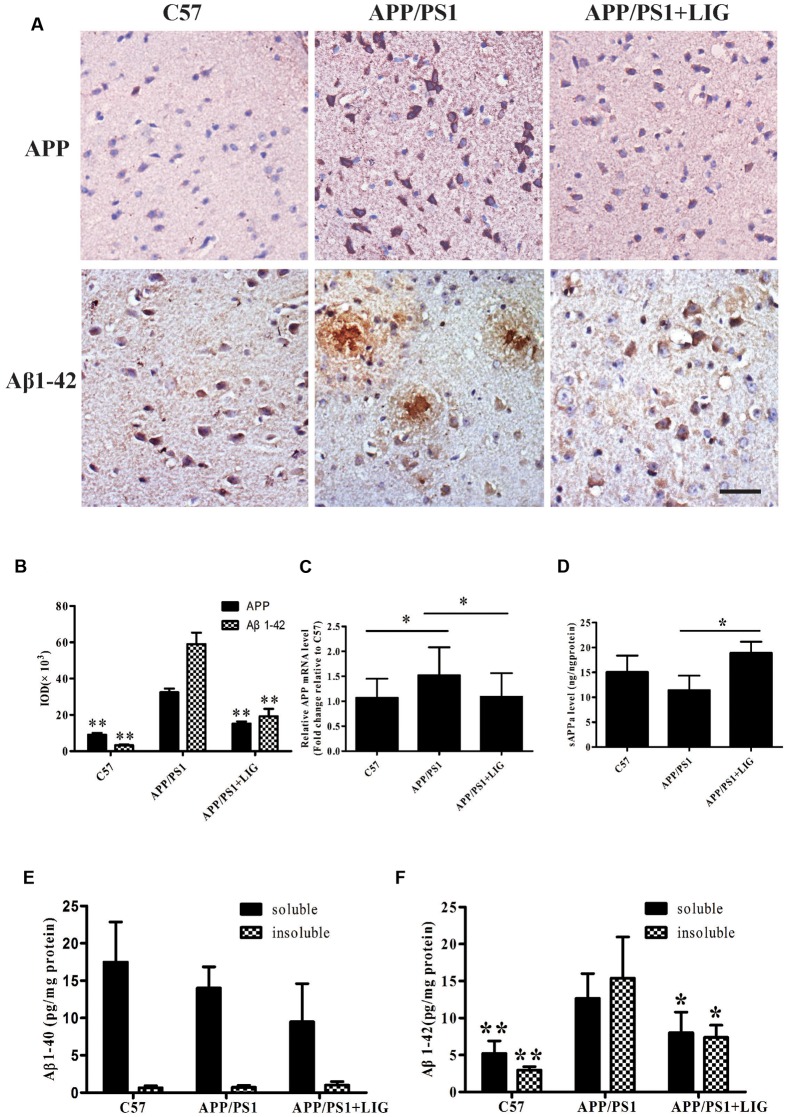
Effect of LIG on APP processing and amyloid-β generation in APP/PS1 transgenic mice. **(A)** Representative immunostaining of APP and Aβ42 in the cerebral cortex of mice treated ± LIG or vehicle control. Scale bar, 25 μm. **(B)** Quantification of immunostaining from **(A)** based on the IOD of positive immunostaining. **(C)** Effect of LIG on full-length APP gene expression in APP/PS1 mice as measured by RT-PCR. **(D)** Effect of LIG on secreted sAPPα in APP/PS1 mice as measured by ELISA. **(E,F)** Effects of LIG on soluble and insoluble Aβ40 peptides **(E)** and Aβ42 peptides **(F)** in brain homogenates of APP/PS1 transgenic mice as measured by ELISA. The data are expressed as mean ± SEM; *n* = 4 for immunohistochemistry and RT-PCR, *n* = 6 for biochemical analysis; ^∗^*p* < 0.05, ^∗∗^*p* < 0.01.

To further study the effect of LIG on APP processing *in vivo*, we examined full-length APP expression and Aβ plaque deposition in the brains of C57 and APP/PS1 mice by immunohistochemistry. **Figure 3A** shows that while there is a significant increase in APP expression and Aβ plaque deposition in the APP/PS1 mice treated with vehicle control compared to C57 mice, chronic administration of LIG significantly rescues this effect, almost to the level of the C57 control mice (*p* < 0.01, quantification in **Figure 3B**). Additionally, compared to C57 mice, APP/PS1 mice show higher levels of full-length APP gene expression and reduced sAPPα secretion. However, treatment with LIG resulted in a 30% reduction in APP gene expression by RT-PCR (*p* < 0.05) and also stimulated the release of sAPPα (*p* < 0.05) (**Figures 3C,D**), suggesting that LIG not only affects APP synthesis but also directs APP processing toward the non-amyloidogenic pathway.

Lastly, we examined the contribution of LIG to Aβ40 and Aβ42 expression in TBS-soluble (soluble) and guanidine-soluble (insoluble) brain fractions. Elevated Aβ42 has been identified as an important event in the early pathogenesis of AD (Findeis, 2007), moreso than the relative expression of Aβ40. This is important to note as we did not see any changes in Aβ40 expression in the APP/PS1 transgenic mouse model used in this study, and yet we found much higher levels of Aβ42 expression in both soluble and insoluble brain fractions of APP/PS1 mice (**Figures 3E,F**). Along these same lines, chronic LIG therapy only slightly reduced the Aβ40 expression found in the soluble brain fraction of APP/PS1 mice compared to vehicle treated controls, an effect which did not reach significance (**Figure 3E**). However, when we examined Aβ42 expression following LIG treatment, we show that it is significantly rescued in both the soluble and insoluble brain fractions compared to control, indicating the relevance and specificity of this compound. Collectively, these data indicate that LIG enhances APP processing through the α-secretase pathway, thus inhibiting production of Aβ42.

### LIG Increases Klotho Expression and α-Secretase Processing in APP/PS1 Transgenic Mice

**FIGURE 4:**
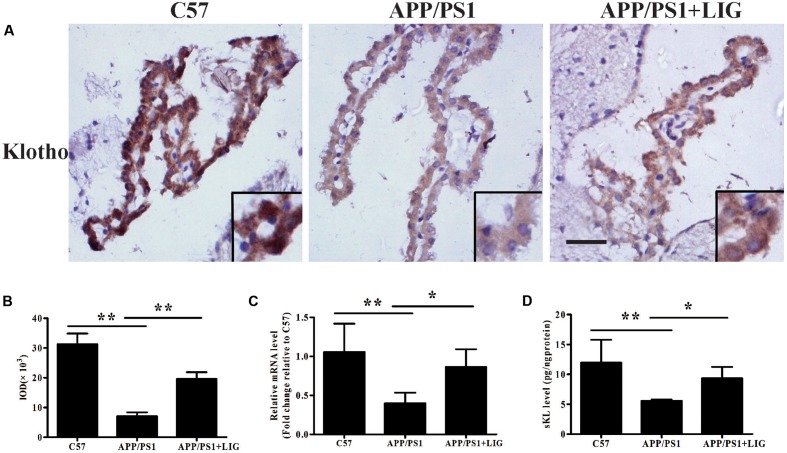
Effect of LIG on Klotho expression and processing in APP/PS1 transgenic mice. **(A)** Representative immunostaining of Klotho in the choroid plexus of APP/PS1 mice in the presence or absence of LIG or vehicle control. Scale bar, 25 μm. **(B)** Quantification of immunostaining from **(A)** based on the IOD of positive immunostaining. **(C)** Effect of LIG on gene expression of Klotho as measured by RT-PCR in APP/PS1 transgenic mice. **(D)** Effect of LIG on sKL secretion as measured by ELISA in APP/PS1 transgenic mice. The data are expressed as mean ± SEM; *n* = 4 for immunohistochemistry and RT-PCR, *n* = 6 for biochemical analysis; ^∗^*p* < 0.05, ^∗∗^*p* < 0.01.

To study the effect of LIG on Klotho processing *in vivo*, we examined its expression in the choroid plexus of mice by immunohistochemistry. **Figure 4A** shows that while there is a significant reduction in Klotho staining in APP/PS1 vehicle-treated mice compared to C57 control mice, LIG significantly reversed this effect; indeed, quantitative analysis of our immunostaining reveals an increase in Klotho expression by more than 100% (*p* < 0.01, **Figure 4B**). Along these same lines, LIG treatment significantly increased Klotho gene expression in APP/PS1 mice as measured by RT-PCR (*p* < 0.05) and also stimulated the release of sKL as measured by ELISA (*p* < 0.05) (**Figures 4C,D**). These results indicate that LIG not only increases expression of full-length Klotho in the brains of APP/PS1 mice, but also increases the production of α-secretase processed sKL, which is then released into circulation.

### Upregulation of Klotho by LIG Leads to an Inhibition of IGF-1/Akt/mTOR Signaling *in Vitro* and *in Vivo*

**FIGURE 5:**
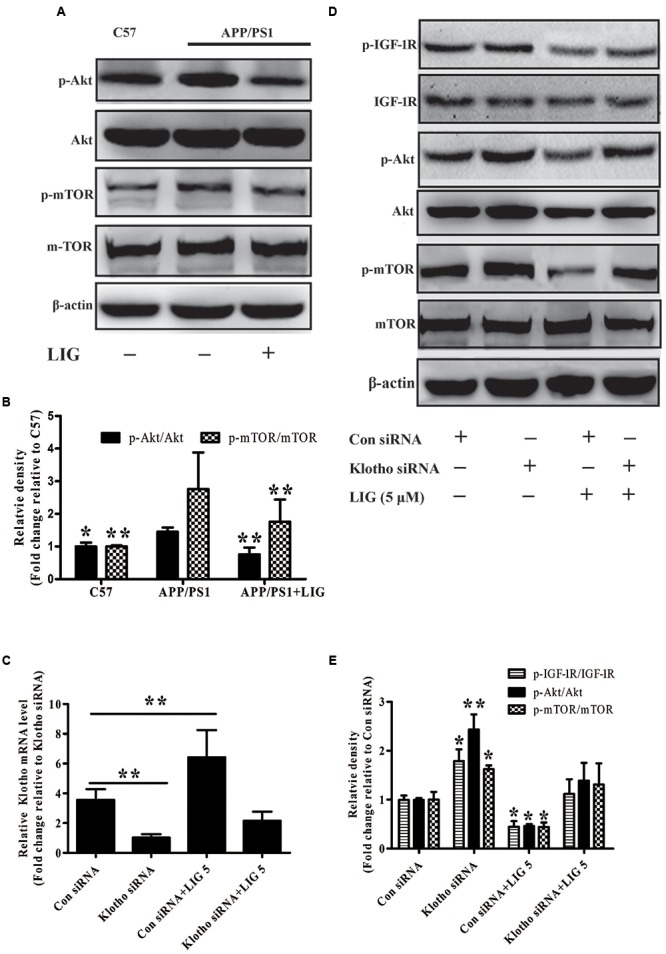
Effect of LIG on insulin growth factor 1 (IGF-1)/Akt/m-TOR signaling pathways *in vivo* and *in vitro.* **(A)** Representative western blot analysis of brain homogenates from C57 or APP/PS1 mice treated ± LIG. **(B)** Quantification of western blot from **(A)** normalized to control C57 mice. **(C)** Full-length Klotho gene expression as measured by RT-PCR in HEK293T cells transfected with either control or Klotho-specific siRNA in the presence or absence of LIG. **(D)** Representative western blot analysis of HEK293T cells from **(C)** indicating the effect of Klotho inhibition on LIG induced IGF-1/Akt/mTOR signaling. **(E)** Quantification of western blot from **(D)** normalized to control C57 mice. The data are expressed as mean ± SEM; *n* = 4 for western blot and RT-PCR; ^∗^*p* < 0.05, ^∗∗^*p* < 0.01.

Accumulation of misfolded Aβ in the brain is believed to be a net result of imbalance between its production and removal (Zuroff et al., 2017). Therefore, we investigated if LIG-mediated Klotho upregulation might have an effect on Aβ clearance. As described previously, Klotho is a transmembrane protein whose cleavage results in the formation of sKL. Once in circulation, sKL acts as a hormone which is able to inhibit the insulin growth factor 1 (IGF-1) signaling pathway, a prominent regulator of stress resistance and aging (Cohen et al., 2009; Abramovitz et al., 2011). The IGF-1 signaling pathway induced activation of PI3K and thereby the Akt/mTOR. Down-regulate mTOR activity permitting activation of autophagy that clears misfolded proteins including Aβ plaques (O’Neill et al., 2012; Zhu et al., 2013). Therefore, we specifically investigated the effect of LIG-mediated Klotho regulation on Akt/mTOR signaling in APP/PS1 mice using western blot analysis. **Figure 5A** shows a significant increase in phosphorylation of both Akt and mTOR in APP/PS1 vehicle treated mice compared to C57 control mice and this was significantly reduced in mice chronically treated with LIG (quantification shown in **Figure 5B**). Next, we asked if the reduction in p-Akt and p-mTOR by LIG is an effect of its ability to regulate Klotho processing, with sKL acting as a ligand for the IGF-1 signaling pathway. Transient transfection of HEK293T cells with an siRNA against Klotho reduced its gene expression by approximately 75% compared to cells transfected with a control siRNA, regardless of LIG treatment (**Figure 5C**). Using western blot analysis 48 h following siRNA transfection, we examined the role of Klotho in Akt/mTOR and IGF-1 signaling in the presence and absence of LIG. Knocking down Klotho gene expression by siRNA significantly increased IGF-1R phosphorylation and increased phosphorylation of Akt and mTOR (**Figures 5D,E**). Interestingly, inhibition of Klotho gene expression resulted in the abrogation of LIG-mediated IGF-1 pathway inhibition, revealing that inhibition of this signaling pathway by LIG is mediated by its upregulation of endogenous Klotho.

## Discussion

The formation of Aβ fibrils is associated with a cascade of neuropathogenic events which induce brain neurodegeneration and lead to the progression of AD (Kirkitadze and Kowalska, 2005). Disruption of the Aβ clearance machinery and an increase in Aβ accumulation gives rise to neurotoxic assemblies. Therapeutic strategies aimed at lowering cerebral Aβ by inhibiting its production and/or enhancing its clearance are currently under development (Lemere, 2009; Kurz and Perneczky, 2011). LIG has been proposed to be a potentially promising candidate for the treatment of AD based on its efficacy shown in our previous studies. But until now, the actual therapeutic value and mechanism of action of LIG on AD pathology and cognitive deficit had not fully been clarified. In the current work, we demonstrated that LIG treatment upregulates the α-secretase processing of APP and Klotho, playing a role in Aβ production and clearance in APP/PS1 transgenic mice.

Previous studies have shown that Aβ plaque deposition occurs before and/or in the early stages of neurodegeneration and behavioral changes in AD patients (Selkoe, 1994). Our results showed that LIG significantly improves the neurobehavioral deficits of APP/PS1 mice in both the passive avoidance and Y-maze tests for working memory. Our studies also showed that LIG treatment decreases aggregated soluble and insoluble Aβ42 levels and also decreases Aβ plaque deposition in the brains of APP/PS1 mice. Based on these results, we hypothesized that the ability for LIG to decrease cerebral Aβ accumulation may be attributable to two effects: directing APP processing toward a non-amyloidogenic pathway thus inhibiting Aβ production, and directing Klotho processing toward releasing sKL and thus acting as a regulator of Aβ clearance. Our studies confirmed that LIG significantly enhances sAPPα release, precluding Aβ generation. sAPPα has been shown to be beneficial for memory function and also to possess both neuroprotective and neurotrophic properties (Mattson, 1997). Results from our study indicate the potential role for LIG-mediated production of sAPPα to serve as a neuroprotective agent and contribute to the long-term benefit of LIG on memory loss in APP/PS1 mice.

Members of the ADAM family are considered likely α-secretase candidates responsible for alpha-cleavage in cells (Vingtdeux and Marambaud, 2012), and ADAM10 and ADAM17 specifically have been considered the likely candidates for α-secretase mediated APP cleavage (Nunan and Small, 2000) and Klotho processing. Results from our study showed that long-term LIG administration promotes non-amyloidogenic processing of APP in addition to sKL processing *in vitro* and *in vivo*, by increasing α-secretase cleavage of full-length APP and Klotho. In the brains of APP/PS1 mice, only ADAM10 protein levels were significantly elevated following LIG treatment, with no effect of LIG on ADAM17. To more fully understand the effect of LIG on ADAM-regulated processing of APP and Klotho, we used the selective, competitive inhibitors G1254023X and TAPI-O against ADAM10 and ADAM17, respectively. We found that ADAM10 inhibition significantly rescued the effect of LIG with no effect of ADAM17 inhibition, indicating the involvement of ADAM10 in LIG-induced APP and Klotho processing.

Apart from inhibition of Aβ production, promotion of Aβ clearance is also regarded as a valuable strategy in treating AD (Wostyn et al., 2011). Recently, it has been shown that impairment of autophagy contributes to an abnormal accumulation of Aβ protein in several age-dependent neurodegenerative diseases, including AD, Huntington’s disease, and Parkinson’s disease. And indeed, several studies have indicated that autophagy is involved in the clearance of Aβ under physiological conditions, effectively maintaining Aβ homeostasis in the healthy brain (Hung et al., 2009; Tian et al., 2011; Li et al., 2013). However, emerging evidence has suggested that autophagy is reduced in the brains of AD patients and animal models of AD, and this reduction leads to the accumulation of Aβ, subsequently contributing to its pathogenesis (Pickford et al., 2008; Nixon and Yang, 2011; Tan et al., 2014). Further evidence has suggested that compounds which inhibit mTOR signaling can increase Aβ clearance and rescue memory impairment by enhancing autophagy in AD mouse models (Spilman et al., 2010; Vingtdeux et al., 2011).

A previous study from our lab showed that Klotho alleviated oxidative stress by negatively regulating IGF-1 signaling pathway and thereby prevented the development of aging-associated AD (Kuang et al., 2014b). Consistent with these results, it has been reported that Klotho-induced mTOR inhibition primarily depends on its ability to inhibit the IGF-1/Akt/mTOR signaling cascade (Lin et al., 2013; Xie et al., 2013). Based on these studies, we speculated that sKL upregulated by LIG might have effect on promoting Aβ clearance via enhancing autophagy through inhibition of the IGF-1/Akt/mTOR pathway, an important pathway associated with Aβ autophagic clearance (Steele and Gandy, 2013). Our results showed that LIG resulted in significant inhibition of Akt/mTOR signaling in APP/PS1 mice as measured by western blot. Furthermore, we revealed the necessity of Klotho for this effect of LIG by target siRNA to knockdown Klotho expression in HEK293T cells.

## Conclusion

Our data demonstrate for the first time that LIG is able to reduce cerebral Aβ burden and improve cognitive impairment in the APP/PS1 transgenic mouse model of AD via enhancing ADAM10 activity. The possible underlying mechanism of LIG may include two aspects: (1) LIG promotes the non-amyloidogenic processing of APP resulting in increased sAPPα levels and decreased Aβ production; (2) LIG upregulates sKL generation with inhibition of IGF-1/Akt/mTOR signaling. Taken together with the reported neuroprotective effects of both sAPPα and sKL as well as autophagy induction by Akt/mTOR pathway inhibition, our findings suggest that LIG may benefit for AD treatment through Aβ generation inhibition and potential clearance promotion via induction alpha-processing of APP and Klotho. Whether LIG may induce Aβ autophagic clearance and the underlying mechanisms need to be further investigated in future. In addition, in order to know the potential effects of LIG on alpha-processing single transmembrane proteins under physiological conditions, a new study with LIG-treated wild-type mice needs to be done in future.

## Author Contributions

Study design: J-RD and XK. Data collection and analysis: XK, H-JZ, X-NC, L-JL, and J-RD. Study supervision: J-RD. Paper drafting: XK, AT, and J-RD. Paper revising: J-RD, XK, and AT. Final approval of the version to be published: All authors.

## Conflict of Interest Statement

The authors declare that the research was conducted in the absence of any commercial or financial relationships that could be construed as a potential conflict of interest.

## Supplementary Material

FIGURE S1 Effect of LIG on cell viability. **(A)** Analysis of cell viability using MTT assay in HEK293T cell after 24 h of incubation with LIG at 0.1–10 μM. **(B)** Analysis of cell viability using MTT assay in SH-SY5Y cells after 24 h of incubation with LIG at 0.1–10 μM. **(C)** Analysis of cell viability using MTT assay in hAPP-transfected SH-SY5Y cells 24 h after transfection. **(D)** Analysis of cell viability using MTT assay in HKE293T cells 24 h after Klotho siRNA transfection.
