# Supplementary figures and images for: Neuroprotective Effect of Ligustilide through Induction of α-Secretase Processing of Both APP and Klotho in a Mouse Model of Alzheimer’s Disease
Source: Front Aging Neurosci. 2017 Nov 2;9:353. doi: 10.3389/fnagi.2017.00353 (PMC5673635; doi:10.3389/fnagi.2017.00353)

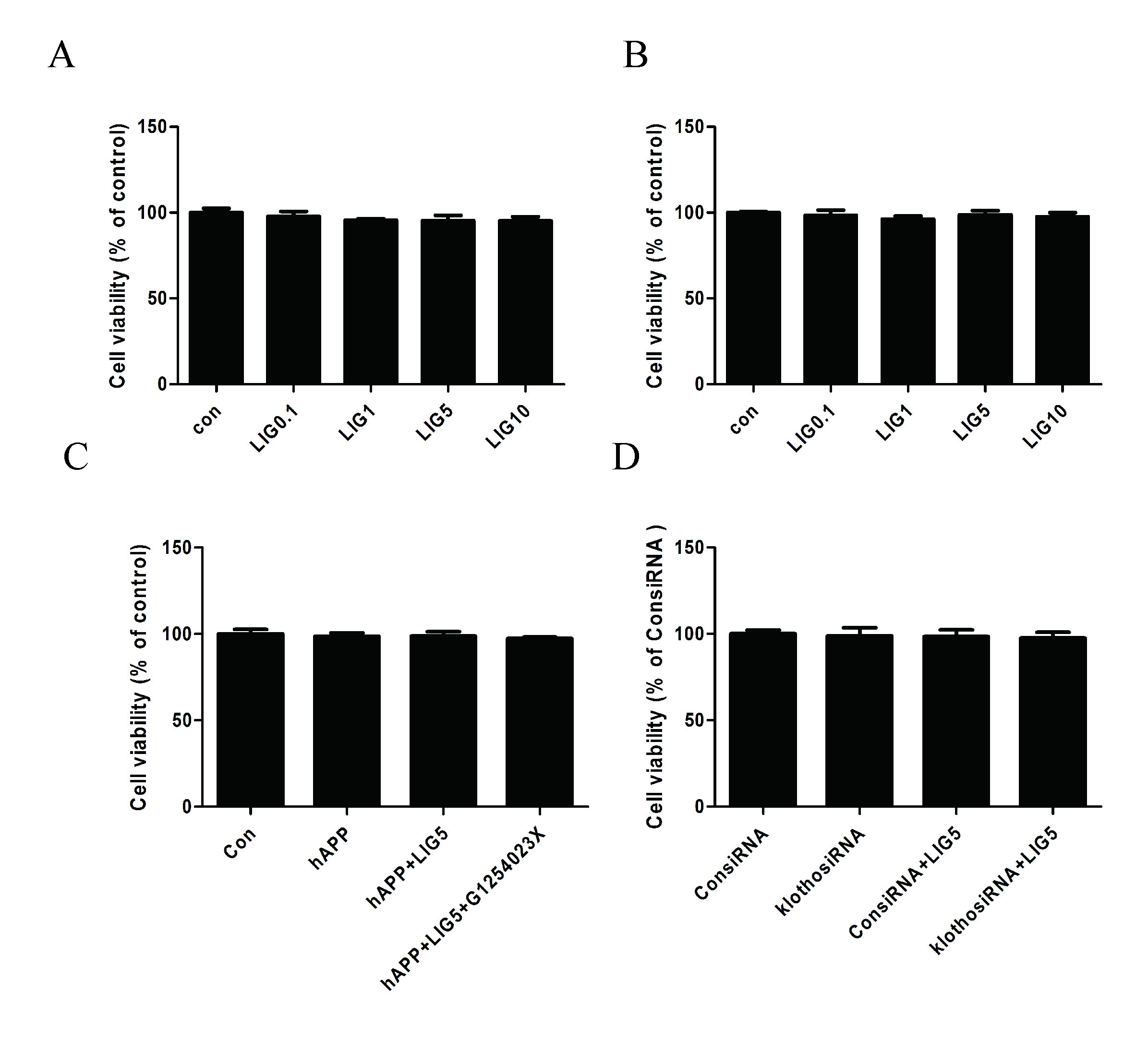

Supplement: FIGURE S1 — Effect of LIG on cell viability. (A) Analysis of cell viability using MTT assay in HEK293T cell after 24 h of incubation with LIG at 0.1–10 μM. (B) Analysis of cell viability using MTT assay in SH-SY5Y cells after 24 h of incubation with LIG at 0.1–10 μM. (C) Analysis of cell viability using MTT assay in hAPP-transfected SH-SY5Y cells 24 h after transfection. (D) Analysis of cell viability using MTT assay in HKE293T cells 24 h after Klotho siRNA transfection. [file Image_1.jpeg]
